# An Improved Voltage Clamp Circuit Suitable for Accurate Measurement of the Conduction Loss of Power Electronic Devices

**DOI:** 10.3390/s21134285

**Published:** 2021-06-23

**Authors:** Qiuping Yu, Zhibin Zhao, Peng Sun, Bin Zhao, Yumeng Cai

**Affiliations:** State Key Laboratory of Alternate Electrical Power System with Renewable Energy Sources, North China Electric Power University, Beijing 102206, China; yuqiuping@ncepu.edu.cn (Q.Y.); sunpeng@ncepu.edu.cn (P.S.); binzhao@ncepu.edu.cn (B.Z.); caiyumeng@ncepu.edu.cn (Y.C.)

**Keywords:** power electronic devices, drain–source voltage clamp circuit, power loss, conduction loss, on-state voltage

## Abstract

Power electronic devices are essential components of high-capacity industrial converters. Accurate assessment of their power loss, including switching loss and conduction loss, is essential to improving electrothermal stability. To accurately calculate the conduction loss, a drain–source voltage clamp circuit is required to measure the on-state voltage. In this paper, the conventional drain–source voltage clamp circuit based on a transistor is comprehensively investigated by theoretical analysis, simulations, and experiments. It is demonstrated that the anti-parallel diodes and the gate-shunt capacitance of the conventional drain–source voltage clamp circuit have adverse impacts on the accuracy and security of the conduction loss measurement. Based on the above analysis, an improved drain–source voltage clamp circuit, derived from the conventional drain–source voltage clamp circuit, is proposed to solve the above problems. The operational advantages, physical structure, and design guidelines of the improved circuit are fully presented. In addition, to evaluate the influence of component parameters on circuit performance, this article comprehensively extracts three electrical quantities as judgment indicators. Based on the working mechanism of the improved circuit and the indicators mentioned above, general mathematical analysis and derivation are carried out to give guidelines for component selection. Finally, extensive experiments and detailed analyses are presented to validate the effectiveness of the proposed drain–source voltage clamp circuit. Compared with the conventional drain–source voltage clamp circuit, the improved drain–source voltage clamp circuit has higher measurement accuracy and working security when measuring conduction loss, and the proposed component selection method is verified to be reasonable and effective for better utilizing the clamp circuit.

## 1. Introduction

High-voltage, large-capacity power electronic conversion equipment dramatically improves the transmission capacity of the flexible AC/DC grid as well as the electric traction control ability [1]. As the voltage withstand ability and switching frequency of power semiconductor devices continue to increase, and the volume continues to decrease, power electronic converters have higher efficiency and power density [2,3,4]. However, converters face reliability challenges. The overheating failure of the internal power electronic devices is one of the main reasons for damage to the converter [5,6,7], and most heat comes from the power loss of power semiconductor devices. Imprecise power loss measurement will lead to the wrong design of the thermal management system (TMS), which will affect the reliability and cause premature failure of the equipment [8,9]. Accurately obtaining the power loss of the device is a crucial prerequisite for determining the thermal solution, which will affect the efficiency, cost, and power density of the entire system.

Common methods for obtaining power loss are calorimetry [10,11], building a physical model [12,13], establishing a loss look-up table or fitting power loss as a function [14,15], and directly integrating the product of the square of the on-state current root mean square and the on-state resistance [16]. However, the above methods often introduce significant errors due to model or measurement problems.

At present, an accurate way to calculate power loss, including switching loss and conduction loss, is to measure the on-state drain–source voltage (*v*_ds_on_) across the device and the current (*i*_d_) flowing through the device and then integrate their product. However, when calculating the conduction loss, it is difficult to measure the on-state voltage accurately. It is directly related to the operating characteristics of the power semiconductor device that frequently converts between the on state and the off state. The off-state drain–source voltage (*v*_ds_off_) can reach hundreds or thousands of volts, while the on-state voltage *v*_ds_on_ is only a few volts [17], making it challenging to select a suitable oscilloscope range. An excessive range will lead to a significant error in the on-state voltage measurement, and more seriously, the measurement result may be negative due to the influence of noise. If the oscilloscope range is set too small, the “oscilloscope saturation” phenomenon will be seen [18].

The drain–source voltage clamp circuit (DVCC) is frequently used to measure the on-state voltage *v*_ds_on_ by clamping the off-state voltage of the device under test (DUT) to a lower value. In existing research, six types of DVCCs have been proposed for the measurement of *v*_ds_on_. The DVCC proposed in [19,20] clamps the off-state voltage of the DUT by using the high-voltage breakdown characteristics of the Zener diode. It is simple to implement. However, its measurement error due to the leakage current of the Zener diode and its measurement delay due to the resistance–capacitance (RC) loop limit its further application. Gelagaev [18] analyzed the DVCC based on a current mirror in detail, and this circuit solved the problem of measurement delay. However, it has been shown that since the output currents on both sides of the current mirror cannot be entirely consistent, the current flowing through the diodes on both sides may be different, which may cause measurement errors. Furthermore, in [21], a DVCC based on one diode is described. The forward voltage of its diode is affected by temperature and current, leading to cumbersome corrections for *v*_ds_on_. This problem was resolved in [22,23]. The DVCC proposed in [22,23] introduced two diodes and a proportional amplifier circuit to improve measurement accuracy. However, due to the difference in the physical positions and forward current of the two diodes, it is difficult to ensure that the voltage drop of the two diodes is equal, which may lead to inaccurate results. Yu et al. [24] presented an innovative design for the DVCC with improved real-time measurement accuracy. Guacci et al. described a DVCC in [25], which can accurately correct the voltage offset caused by the diode voltage drop and has a higher measurement accuracy. Both of these circuits in [24,25] introduce operational amplifiers, which increases the complexity of the course. A DVCC integrated with a half-bridge circuit was employed in [26] for device evaluation in the hard-switching test and the soft-switching conditions. However, this DVCC ignores the influence of diode leakage current on the on-state voltage measurement, and there may be measurement errors. The DVCC based on the transistor is analyzed in [27]. It avoids most of the problems mentioned earlier. However, the gate–source spike voltage due to the instantaneous high current of the transistor gate resistance may damage the DVCC itself. Additionally, the purpose of designing this circuit is to measure on-state resistance *R*_ds_on_, and it is not suitable for conduction loss measurement.

In summary, these existing DVCCs, in terms of measurement accuracy, work complexity, and design aim, cannot be used for conduction loss (*P*_loss_on_) measurement of power semiconductor devices. Therefore, this paper concerns the drawbacks of the conventional DVCC based on the transistor (hereinafter referred to as “conventional DVCC”) and proposes an improved DVCC (hereinafter referred to as “improved DVCC”) architecture suitable for the *P*_loss_on_ measurement. The new circuit is derived from the conventional DVCC. The remainder of this paper is organized as follows. In Section 2, the circuit structure, work principle, and drawbacks of the conventional DVCC are analyzed in detail. Then, the schematic and the advantages of the improved DVCC are presented. Furthermore, the influence of the components’ parameters on the circuit performance of the improved DVCC is analyzed in Section 3. Here, component selection guidelines are also given. In Section 4, the measurement accuracy and work security improvement of the improved DVCC are verified through simulated and experimental comparisons with the conventional DVCC. Simultaneously, the effectiveness of the selection theory is also investigated and proven. Finally, Section 5 concludes this paper.

## 2. Design of the Improved DVCC

Compared with the existing DVCCs, which are mainly applied to the on-state resistance measurement of the DUT, more problems need to be considered when designing a DVCC suitable for conduction loss measurement. After the DUT is turned on, it will go through two typical states, enter the oscillation state (on-oscillation state), and gradually reach steady state (on-steady state). The measurement results of these existing DVCCs can well reflect the on-state voltage *v*_ds_on_ste_ when the DUT is in the on-steady state. However, when measuring the conduction loss, in addition to the above-mentioned on-steady state voltage, the DVCC must be able to accurately measure the on-state voltage *v*_ds_on_osc_ when the DUT is in the on-oscillation state. Any error at any stage will cause errors in the calculation of the device conduction loss.

In addition, many common problems need to be avoided in both on-state resistance and conduction loss measurement. First of all, the DVCCs cannot have measurement delays. Once the voltage data lag or lead the current data, errors will occur in the loss integral calculation. Secondly, power electronic devices, such as diodes, metal–oxide–semiconductor field–effect transistors (MOSFET), etc., are often introduced into DVCCs to realize the voltage clamping function. Due to the faster switching speed and higher operating voltage of the DUT, it is easy to make these auxiliary devices out of safe working conditions. Therefore, when designing a DVCC, it is necessary to focus on the security of these auxiliary devices.

### 2.1. The Structure and Working Principle of the Conventional DVCC

**Figure 1:**
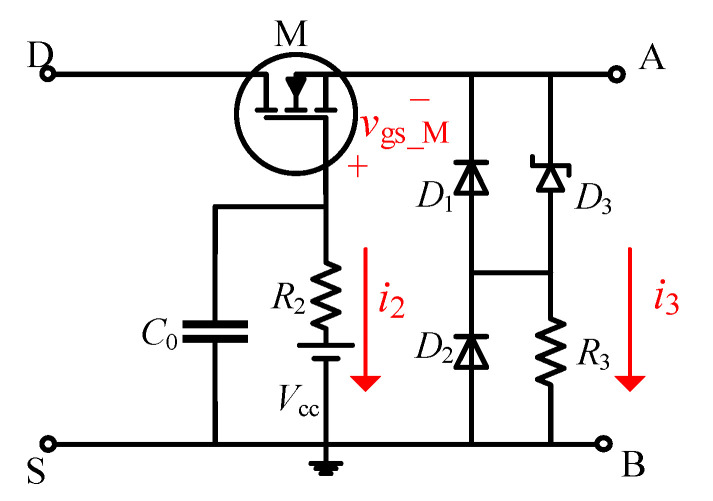
The schematic diagram of the conventional DVCC.

The schematic diagram of the conventional DVCC is shown in Figure 1. The auxiliary device MOSFET (M) is used to withstand the high off-state voltage of the DUT, thereby limiting the potential of the voltage measurement point A. The DC voltage supply *V*_cc_ and the gate resistor *R*_2_ are located at the gate of M, and together with the resistor *R*_3_, they control the turn-on and turn-off of M. The D and S terminals of the circuit are connected to the drain and source of the DUT, respectively, while the A and B terminals are used to measure output voltage (*v*_out_).

When the DUT is in the off-state, *D*_3_ is broken down, causing the current flowing through *R*_3_ to increase sharply. At this time, the source potential of M rises, and the gate-source voltage *v*_gs_M_ decreases. When *v*_gs_M_ is less than the threshold voltage *V*_th_M_ of M, M is turned off and shares most of the off-state voltage, limiting *v*_out_ to a small voltage value. When the DUT is turned on, the source potential of M decreases, causing the *v*_gs_M_ to become higher than *V*_th_M_, bringing the M into conduction. As a result, *v*_out_ is equal to *v*_ds_on_.

### 2.2. Drawbacks of the Conventional DVCC

In view of the fact that the purpose of the conventional DVCC is to measure the on-state resistance, when it is applied to conduction loss measurement, there are some severe problems, which are further discussed in the following subsections.

#### 2.2.1. Low Measurement Accuracy

To reduce the voltage negative overshoot between A and B when the DUT is turned on, the conventional DVCC connects the diodes *D*_1_ and *D*_2_ in reverse parallel between the measurement points A and B and utilizes their unidirectional conductivity characteristics to eliminate the voltage overshoot.

Assume that the forward voltage drops of diodes are *V*_D1_ and *V*_D2_, respectively. During the on-oscillation state, if *v*_ds_on_osc_ is greater than −(*V*_D1_ + *V*_D2_), *D*_1_ and *D*_2_ are reversely cut off. Therefore, *v*_out_ = *v*_ds_on_osc_. Once *v*_ds_on_osc_ is less than −(*V*_D1_ + *V*_D2_), *D*_1_ and *D*_2_ will immediately switch to the forward conduction state, and the output voltage will remain unchanged at −(*V*_D1_ + *V*_D2_), resulting in *v*_out_ ≠ *v*_ds_on_. The two diodes limit the negative voltage overshoot and do not affect the on-state resistance measurement. However, when measuring the conduction loss, the loss during oscillation cannot be ignored [28]. The conventional DVCC cannot measure *v*_ds_on_osc_ accurately nor can it accurately measure the conduction loss.

#### 2.2.2. Low Working Security

To reduce the current flowing through the DC voltage supply (*V*_cc_) and ensure the safety of *V*_cc_, the conventional DVCC shown in Figure 1 has a capacitor *C*_0_ connected in parallel to the gate of M [22]. However, the existence of *C*_0_ seriously affects the work security of auxiliary device M. When the DUT is turned off, the drain–source voltage *v*_ds_ and the current on *R*_3_ increases sharply, which will cause an instantaneous negative overshoot *V*_gs_M(max)_ at the gate–source of M. If *V*_gs_M(max)_ exceeds the gate−source voltage withstand limit of M (*V*_gs_limit_), M will be burned. If negative overshoot *V*_gs_M(max)_ occurs at time *t*_0_, the gate−source voltage *v*_gs_M_ of M will be a negative value in the time interval [*t*_0_ − Δ*t*, *t*_0_ + Δ*t*]. According to Figure 1, it can be known from KVL that during this period, *v*_gs_M_ can be written as in Equation (1), and *V*_gs_M(max)_ = *v*_gs_M_(*t*_0_):

(1) $$v_{\mathrm{gs}\_M}{=V}_{\mathrm{cc}}{+i}_{2}R_{2}-V_{D3}-i_{3}R_{3},$$

where *V*_D3_ is the breakdown voltage of Zener diode *D*_3_; *i*_2_ and *i*_3_ are the current flowing through *R*_2_ and *R*_3_, respectively.

Adding *C*_0_ to the gate of M will reduce *i*_2_(*t*_0_), which can protect *V*_cc_. However, since *V*_gs_M(max)_ is negative, the decrease in *i*_2_(*t*_0_) will cause the absolute value of *V*_gs_M(max)_ to increase significantly, which will endanger the safety of the MOSFET. In contrast, the impulse current withstand capability of the widely used DC voltage supply can reach several amperes or tens of amperes. Even without *C*_0_, *i*_2_(*t*_0_) is not enough to cause harm to *V*_cc_. Therefore, the benefit of *C*_0_ is far less than the harm it causes.

### 2.3. Proposal of the Improved DVCC

**Figure 2:**
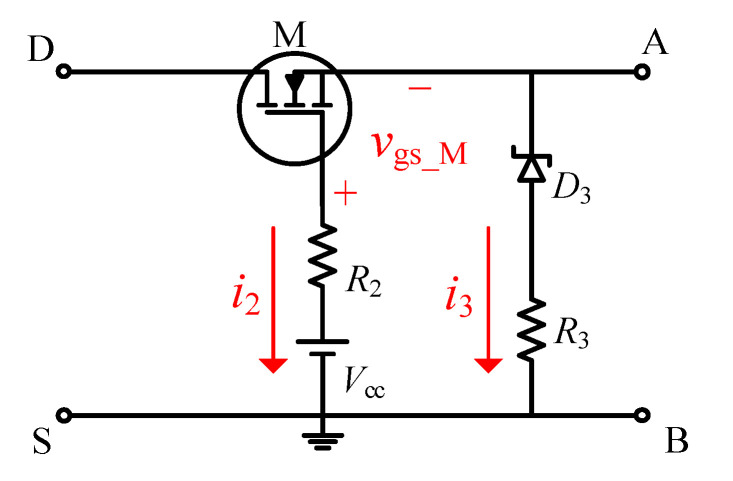
The schematic diagram of the improved DVCC.

The schematic of the improved DVCC for conduction loss measurement is described in Figure 2. By conducting two changes in the structure of the conventional DVCC, the problems existing in the conventional DVCC are solved.

Firstly, to accurately measure *v*_ds_on_osc_ of the DUT, the improved DVCC removes anti-parallel diodes (i.e., *D*_1_ and *D*_2_ in Figure 1) from the output voltage measurement point. Under this arrangement, the on-state voltage of the two stages, on-steady state and on-oscillation state, both have high measurement accuracy. Furthermore, the calculation error of conduction loss is limited to a small value.

In addition, considering the harmfulness of *C*_0_ to the core device M, another improvement is to remove the gate-shunt capacitance (i.e., *C*_0_ in Figure 1). This measure dramatically improves the operating environment of M. In addition, there is no need to worry about the safety of *V*_cc_. For typical DC sources, their impulse current tolerance often reaches several amps or tens of amps, while the maximum current flowing through *V*_cc_ is usually hundreds of milliamps. Therefore, the work safety of *V*_cc_ will not be threatened.

## 3. Component Selection

In Section 2, the pros of the improved DVCC and the cons of conventional DVCC were highlighted. In this section, the influence of component parameters on the performance of the improved DVCC is analyzed in detail. Furthermore, guidelines for component selection are given to utilize the improved circuit better.

### 3.1. Evaluation Indicators

Theoretically, there are three conditions that the circuit must meet to perform the functions of clamping and measuring normally, as listed below.

1. Ensure the security of core MOSFET (M);
2. M should be in the proper working state when the DUT is in the on-state;
3. M should be in the proper working state when the DUT is in the off-state.

Considering the above three restrictions, this article comprehensively extracts three electrical quantities as the judgment indicators of the circuit performance to guide component selection:

1. Gate−source voltage negative overshoot (*V*_gs_M(max)_) of M, which is denoted as *EI*_1_. As indicated in Section 2, it is necessary to avoid *V*_gs_M(max)_ exceeding the gate–source tolerance of core M. Therefore, the low *EI*_1_ value is of greater significance for improving the security of M.
2. Gate−source voltage of M (*v*_gs_M(on)_) when the DUT is in the on-state, which is denoted as *EI*_2_. If the DUT is in the on-state, M should also be in the on-state to satisfy *v*_out_ = *v*_ds_on_. Therefore, the second evaluation indicator should meet *EI*_2_ > *V*_th_M_.
3. Gate−source voltage of M (*v*_gs_M(off)_) when the DUT is in the off-state, which is denoted as *EI*_3_. When the DUT is in the off-state, the working state of M should also be consistent with the DUT to withstand high off-voltage and reduce the potential of the measurement point A. Under this condition, *EI*_3_ should be less than *V*_th_M_, so that M can be turned off reliably [29].

### 3.2. Selection of MOSFET

Since the parameters of M are closely related to the safe operation of the entire circuit, criteria for selecting the subject are proposed based on the working principle of the improved DVCC.

As stated before, with the transitions of DUT from the on-state to the off-state, M also changes its state rapidly so as to prevent the continuous increase of the source current of M and prevent *EI*_1_ from being too large. Similarly, when the DUT changes from the off-state to the on-state, M needs to be turned on immediately to avoid measurement delay. Therefore, it is recommended that the switching speed of M be consistent with or faster than DUT, which is the first criterion for the selection of M.

In addition, since the improved DVCC utilizes the high blocking voltage characteristic of M to exercise the clamping function, during the off-state of the DUT, the drain–source withstand voltage of M is almost the same as that of the DUT. To increase the operational reliability of M, it is advised that the blocking voltage level of M is consistent with or higher than the DUT, which is considered the second criterion.

### 3.3. Selection of DC Voltage Supply V_cc_ and Zener Diode Breakdown Voltage V_D3_

#### 3.3.1. Selection Principle of *V*_D3_

Since Zener diode *D*_3_ is in the gate−source loop of M, *EI*_1_ is one of the vital evaluation indicators for selecting *V*_D3_. The influence of *V*_D3_ on the work security of M is analyzed in this subsection.

According to Figure 2, the gate−source negative overshoot of M can be expressed as follows:

(2) $${EI}_{1}{=V}_{\mathrm{cc}}{+I}_{2}R_{2}-V_{D3}-I_{3}R_{3},$$

where *I*_2_ and *I*_3_ are the currents flowing through *R*_2_ and *R*_3_ at time *t*_0_, respectively.

According to Equation (2), *V*_D3_ increases, and the absolute value of *EI*_1_ increases accordingly. Based on the interpretation content of the first evaluation indicator, for high security of M, *V*_D3_ should be as small as possible.

#### 3.3.2. Voltage Constraint for Effective Work

The DC voltage supply *V*_cc_ and the Zener diode *D*_3_ jointly control the turn-on and turn-off of M to make it follow the steps of the state change of DUT. Therefore, it can be seen that the values of *v*_gs_M(on)_ and *v*_gs_M(off)_ are closely related to *V*_cc_ and *V*_D3_. Based on the supplementary content when the second and third evaluation indicators are proposed, it can be estimated that *V*_cc_ and *V*_D3_ have a mutually restrictive relationship. In this paper, this specific constraint is called the “voltage constraint for effective work (VCEW)” and is further discussed in the subsequent sections.

When the DUT is in the on-state, *EI*_2_ can be expressed as in the equation below:

(3) $${EI}_{2}{=V}_{\mathrm{cc}}{+i}_{2\_\mathrm{on}}R_{2}-v_{\mathrm{ds}\_\mathrm{on}},$$

where *i*_2_on_ is the current flowing through the resistor *R*_2_ when the DUT is in the on-state.

Since M is also in the on-state, *i*_2_on_ can be obtained as follows:

(4) $$i_{2\_\mathrm{on}}{=i}_{g\_M}\;\approx \;0,$$

where *i*_g_M_ is the gate current of M.

Therefore, Equation (3) can be simplified to

(5) $${EI}_{2}{=V}_{\mathrm{cc}}-v_{\mathrm{ds}\_\mathrm{on}}.$$

If the working condition of the DUT is known, the maximum on-state voltage *V*_on_max_ of the DUT is determined. At this time, the size of *EI*_2_ depends on the value of the DC voltage supply (*V*_cc_). To ensure that M is in the on-state, it should meet the following condition:

(6) $$V_{\mathrm{cc}}{\;>\;V}_{\mathrm{on}\_\max}{+V}_{\mathrm{th}\_M}.$$

When the DUT is in the off-state, *EI*_3_ can be described as follows:

(7) $${EI}_{3}{=V}_{\mathrm{cc}}{+i}_{2\_\mathrm{off}}R_{2}-V_{D3}\prime -i_{3\_\mathrm{off}}R_{3},$$

where *i*_2_off_ and *i*_3_off_ are the currents flowing through *R*_2_ and *R*_3_, respectively, when the DUT is in the off-state; *V*_D3_′ is the voltage across the Zener diode *D*_3_.

Since M is in the off-state, *i*_2_off_ and *i*_3_off_ can be obtained as follows:

(8) $$i_{3\_\mathrm{off}}{=i}_{\mathrm{leak}\_M\;}\approx \;0,$$

(9) $$i_{2\_\mathrm{off}}{=i}_{g\_M}\;\approx \;0,$$

where *i*_leak_M_ is the leakage current of M.

Equation (7) is further simplified to

(10) $${EI}_{3\;}{=V}_{\mathrm{cc}}{{-V}_{D3}}^{\prime }.$$

When the DUT is in the off-state, *D*_3_ has two possible scenarios [30]. If the leakage current of *D*_3_ is more significant than M, *D*_3_ is in the reverse cut-off state, and *V*_D3_′ meets the condition:

(11) $$V_{D3}{}^{\prime }\;\leq {\;V}_{D3}.$$

In this scenario, a voltage equilibrium will be established: as *V*_cc_ changes, *V*_D3_′ changes accordingly, so that *EI*_3_ is always maintained at a voltage less than *V*_th_M_. According to Equation (11), when selecting *V*_cc_, its value should satisfy the following condition:

(12) $$V_{\mathrm{cc}}-V_{D3}{\;<\;V}_{\mathrm{th}\_M}.$$

In another scenario, if the leakage current of *D*_3_ is less than M, *D*_3_ is in the breakdown state and *V*_D3_′ = *V*_D3_. Obviously, in this circumstance, *V*_cc_ is selected based on the below equation:

(13) $${EI}_{3}{=V}_{\mathrm{cc}}-V_{D3}{\;<\;V}_{\mathrm{th}\_M}.$$

Considering Equations (6), (12), and (13) and adding in a margin of error, Equation (14) is written to reveal the mechanism of VCEW.

(14) $$\{\begin{array}{c} V_{\mathrm{cc}}{>V}_{\mathrm{on}\_\max}{+V}_{\mathrm{th}\_M}\\ V_{\mathrm{cc}}{-V}_{D3}\;<\;0 \end{array}.$$

### 3.4. Selection of Gate Resistance R_2_ and Source Resistance R_3_

#### 3.4.1. Selection Principle of *R*_2_ and *R*_3_

*R*_2_ and *R*_3_ are located in different branches of the gate−source loop of M, so that they have the opposite effect on *EI*_1_ (*V*_gs_M(max)_). Therefore, by choosing appropriate *R*_2_ and *R*_3_ values, the gate−source voltage negative overshoot (*V*_gs_M(max)_) of M can be suppressed as much as possible.

**Figure 3:**
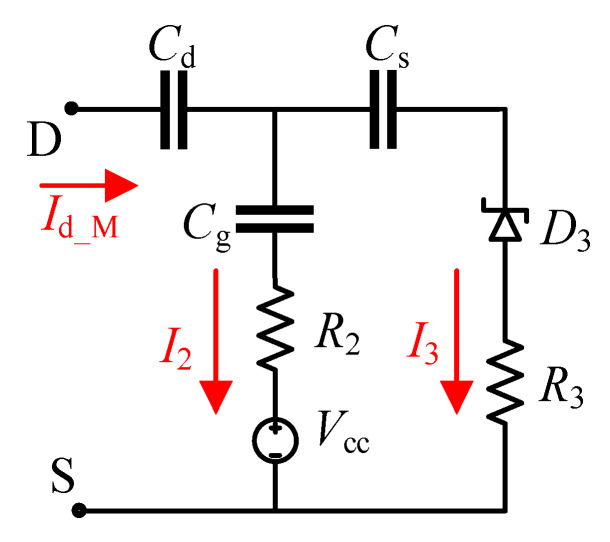
Equivalent circuit of the improved DVCC.

At *t*_0_, when the *V*_gs_M(max)_ occurs, M has been completely turned off, and the improved DVCC can be equivalent to the course shown in Figure 3 [29].

According to Figure 3, the first evaluation indicator can be expressed as

(15) $${EI}_{1}{=V}_{\mathrm{cc}}-V_{D3}+\frac{a(b-jc)}{b^{2}{+c}^{2}}I_{d\_M},$$

(16) $$\{\begin{array}{c} {a=R}_{2}C_{g}-R_{3}C_{s},\\ {b=C}_{g}{+C}_{s},\\ {c=wC}_{s}C_{g}R_{2}{+wC}_{s}C_{g}R_{3}, \end{array}$$

where *I*_d_M_ is the drain current of M.

According to [29], at this time, *C*_g_ << *C*_s_. The coefficient of the third term in Equation (15) is abbreviated as *e* + *jf*. Then, the real and imaginary parts of *EI*_1_ can be written as in Equations (17) and (18), respectively;

(17) $${Re(EI}_{1})=\left|{eI}_{d\_M}\right|+\left|V_{\mathrm{cc}}-V_{D3}\right|,$$

(18) $${Im(EI}_{1})=j\left|{fI}_{d\_M}\right|,$$

(19) $$\left|e\right|=\left|\frac{{(R}_{2}C_{g}{-R}_{3}C_{s}{)(C}_{s}{+C}_{g})}{{{(C}_{g}{+C}_{s})}^{2}{+(wC}_{s}C_{g}R_{2}{+wC}_{s}C_{g}R_{3}{)}^{2}}\right|,$$

(20) $$\left|f\right|=\left|\frac{{(R}_{2}C_{g}{-R}_{3}C_{s}{)(wC}_{s}C_{g}R_{2}{+wC}_{s}C_{g}R_{3})}{{{(C}_{g}{+C}_{s})}^{2}{+(wC}_{s}C_{g}R_{2}{+wC}_{s}C_{g}R_{3}{)}^{2}}\right|.$$

Since the parasitic capacitance of MOSFET is pF level (10^−12^), and the oscillation frequency of drain current is generally MHz level (10^6^~10^8^). Therefore, (*C*_g_ + *C*_s_) >> (*wC*_s_*C*_g_*R*_2_ + *wC*_s_*C*_g_*R*_3_). Based on this, Equations (21) and (22) can be derived;

(21) |e| ≫ |f|,

(22) $$\left|{EI}_{1}\right|\;\approx \;\left|e\right|\;\approx \;\left|\frac{{(R}_{2}C_{g}{-R}_{3}C_{s}{)(C}_{s}{+C}_{g})}{{{(C}_{g}{+C}_{s})}^{2}}\right|=\frac{R_{3}C_{s}{-R}_{2}C_{g}}{C_{g}{+C}_{s}}.$$

According to Equation (22), |*EI*_1_| is positively correlated with *R*_3_ and negatively correlated with *R*_2_. Therefore, the selection guide for these two resistors is to increase *R*_2_ and decrease *R*_3_ as much as possible. It is worth noting that this increase or decrease is not unlimited, which is described in more detail in the following subsection.

#### 3.4.2. Measurement Error Constraint

During the on-oscillation state, with *R*_3_ decreasing, the measurement accuracy of the on-state voltage *v*_ds_on_osc_ gradually decreases. To make the relative error of the *v*_ds_on_osc_ measurement less than *r*%, *R*_3_ cannot be too small. This paper refers to this constraint relationship as the “measurement error constraint” (MEC).

During the on-oscillation state, *v*_ds_on_osc_ can be described as follows:

(23) $$v_{\mathrm{ds}\_\mathrm{on}\_\mathrm{osc}}{=v}_{\mathrm{ds}\_M}{+v}_{\mathrm{out}},$$

where *v*_ds_M_ is the drain–source voltage of M.

During this process, *v*_ds_on_osc_ gradually shifts from the on-oscillation state to the on-steady state in the form of a second-order oscillation. According to the structure of the improved DVCC, *D*_3_ is connected in reverse between A and B. Therefore, when *v*_ds_on_osc_ > 0, *D*_3_ is in the reverse cut-off state, and *v*_out_ can be expressed as follows:

(24) $$v_{\mathrm{out}}{=v}_{\mathrm{ds}\_\mathrm{on}\_\mathrm{osc}}\times \frac{R_{D3\_\mathrm{off}}{+R}_{3}}{R_{D3\_\mathrm{off}}{+R}_{3}{+R}_{\mathrm{ds}\_M}},$$

where *R*_D3_off_ is the equivalent resistance of *D*_3_ when it is in the reverse cut-off state; *R*_ds_M_ is the equivalent resistance of M.

Since M has been fully turned on at this stage, *R*_ds_M_ has the same value as the on-state resistance of M. In addition, considering that *D*_3_ can be regarded as an open circuit at this time, the relationship between *v*_out_ and *v*_ds_on_osc_ can be expressed as follows:

(25) $$v_{\mathrm{out}}\;\approx {\;v}_{\mathrm{ds}\_\mathrm{on}\_\mathrm{osc}}.$$

However, when *v*_ds_on_osc_ < 0, *D*_3_ is in the forward conduction state, and *v*_out_ can be expressed as follows:

(26) $$v_{\mathrm{out}}{=v}_{\mathrm{ds}\_\mathrm{on}\_\mathrm{osc}}\times \frac{R_{D3\_\mathrm{on}}{+R}_{3}}{R_{D3\_\mathrm{on}}{+R}_{3}{+R}_{\mathrm{ds}\_M}},$$

where *R*_D3_on_ is the equivalent resistance of *D*_3_ when it is in the forward conduction state. 

In this case, it is essential that the resistance of *R*_3_ not be too small, so that the measurement accuracy is not compromised due to the partial voltage of *R*_ds_M_. Therefore, restricted by MEC, *R*_3_ needs to meet the following condition:

(27) $$v_{\mathrm{ds}\_\mathrm{on}\_\mathrm{osc}}\times (1-\frac{R_{D3\_\mathrm{on}}{+R}_{3}}{R_{D3\_\mathrm{on}}{+R}_{3}{+R}_{\mathrm{ds}\_M}}{)\;<\;v}_{\mathrm{ds}\_\mathrm{on}\_\mathrm{osc}}\times r\%.$$

Furthermore, Equation (27) is simplified to

(28) $$R_{3}{\;>\;R}_{\mathrm{ds}\_M}\;\times \;(\frac{1-r\%}{r\%})-R_{D3\_\mathrm{on}},$$

where *r*% is generally around 5%.

#### 3.4.3. Switching Speed Constraint

*R*_2_ is located at the gate of M. Therefore, when *R*_2_ increases, the gate charging and discharging speed of the gate driver are slowed down accordingly [29]. In an attempt to ensure that the switching speed of M is not slower than that of the DUT, *R*_2_ cannot be too large. This paper calls this constraint relationship the “switching speed constraint” (SSC).

In order to obtain the limit of *R*_2_, the influence of *R*_2_ on the rising speed of *v*_gs_M_ is simplified as the influence of *R*_2_ on the charging time constant when M is turned on. According to Equations (5) and (10), when M is turned on, the amount of change in *v*_gs_M_ is ((*V*_cc_ − *v*_ds_on_) − (*V*_cc_ − *V*_D3_′)).

Therefore, restricted by SSC, *R*_2_ needs to meet the following condition:

(29) $$\frac{{(V}_{\mathrm{cc}}{-v}_{\mathrm{ds}\_\mathrm{on}}{)-(V}_{\mathrm{cc}}{{-V}_{D3}}^{\prime })}{A}\;<\;\frac{V_{g\_\max}{-V}_{\mathrm{th}\_\mathrm{DUT}}}{B},$$

(30) $$\{\begin{array}{c} A=\frac{1}{R_{2}C_{\mathrm{gs}\_M}}\\ B=\frac{1}{R_{g}C_{\mathrm{gs}\_\mathrm{DUT}}} \end{array}$$

where *V*_g_max_ is the gate−source voltage stability value of the DUT; *R*_g_ and *V*_th_DUT_ are the gate drive resistance and the threshold voltage of the DUT, respectively; and *C*_gs_M_ and *C*_gs_DUT_ are the gate−source parasitic capacitances of M and DUT, respectively.

Considering that *V*_D3_′ ≤ *V*_D3_, Equation (30) can be further simplified to

(31) $$R_{2}<\frac{R_{g}C_{\mathrm{gs}\_\mathrm{DUT}}{(V}_{g\_\max}{-V}_{\mathrm{th}\_\mathrm{DUT}})}{C_{\mathrm{gs}\_M}{((V}_{\mathrm{cc}}{-v}_{\mathrm{ds}\_\mathrm{on}}{)-(V}_{\mathrm{cc}}{-V}_{D3}))}.$$

## 4. Simulation and Experimental Verification

**Figure 4:**
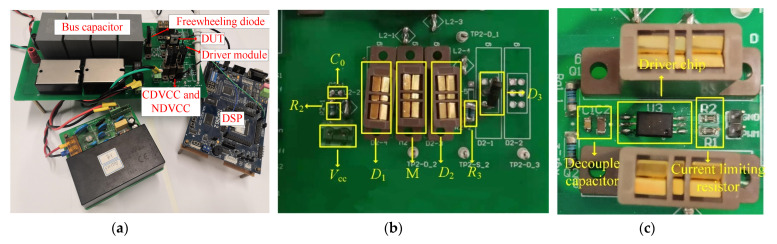
Test platform. (**a**) Test platform overview; (**b**) DVCC section; (**c**) gate driver.

This paper set up a test platform integrating conventional DVCC and improved DVCC, as shown in Figure 4, to evaluate the measurement accuracy and work safety of the improved DVCC and the correctness of the selection theory. The primary circuit of the test platform is a double pulse test circuit (DPTC), including a DUT, freewheeling diode *D*_0_, bus capacitor *C*_bus_, digital signal processing (DSP), and drive module *V*_g_. DSP is used to transmit drive signals to control the turn-on and turn-off of the DUT.

The voltage clamp circuit comprises a MOSFET (M), DC source *V*_cc_, gate resistance *R*_2_, source resistance *R*_3_, and Zener diode *D*_3_. In order to facilitate the comparison between the improved DVCC and conventional DVCC, the connectors for the gate-shunt capacitor *C*_0_ and the anti-parallel diodes *D*_1_ and *D*_2_ are reserved.

The specific experimental conditions are listed in Table 1. Both the DUT and the auxiliary device M are the 1200 V/31.6 A SiC MOSFET produced by CREE, while the freewheeling diode *D*_0_ is the SiC Schottky diode of the unified manufacturer.

**Figure 5:**
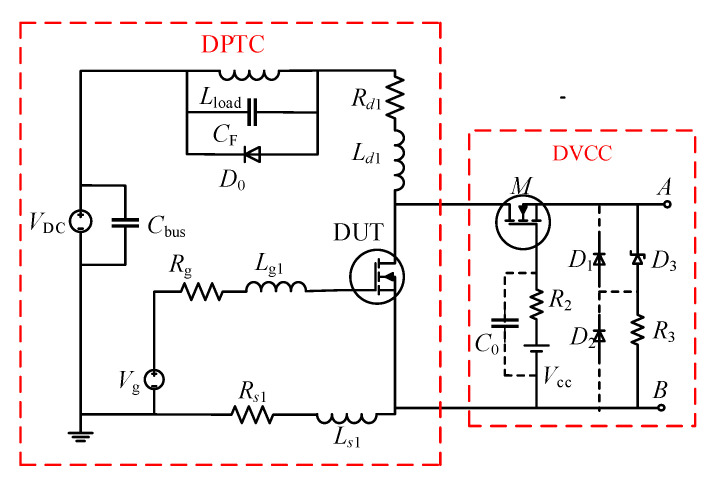
Equivalent simulation circuit.

According to the experimental platform shown in Figure 4, the corresponding equivalent simulation circuit is extracted, as shown in Figure 5. Inside the dotted frame on the right is the DVCC, while the double pulse circuit is in the dotted frame on the left, and its circuit components are shown in Table 2. The simulation models of DUT, M, and *D*_0_ are all from the semiconductor company that produces the device, and the parasitic parameters are extracted by finite element simulation software.

### 4.1. Conduction Loss Measurement Accuracy

#### 4.1.1. Quantitative Simulation Analysis

**Figure 6:**
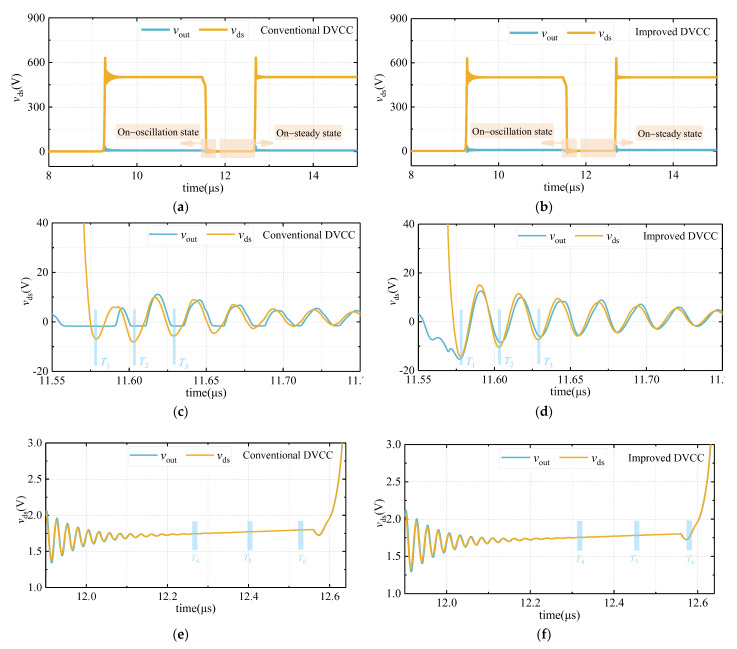
*v*_out_ and *v*_ds_ waveform comparison. (**a**) Waveform overview of the conventional DVCC; (**b**) waveform overview of the improved DVCC; (**c**) waveform of the conventional DVCC during the on-oscillation state; (**d**) waveform of the improved DVCC during the on-oscillation state; (**e**) waveform of the conventional DVCC during the on-steady state; (**f**) waveform of the improved DVCC during the on-steady state.

Since the oscilloscope cannot measure the accurate value of the on-state voltage *v*_ds_on_, it is hard to quantitatively analyze the relative error between the output voltage *v*_out_ and *v*_ds_on_ through experiments. Therefore, this work uses the simulation circuit shown in Figure 5 to compare the on-state voltage and conduction loss measurement accuracy between the conventional DVCC and the improved DVCC. Under the working condition that *V*_DC_ is set to 500 V, the simulation results are shown in Figure 6.

In Figure 6, *v*_ds_ is the voltage waveform measured directly at the drain and source of the DUT. When the DUT is in the on state, *v*_ds_ = *v*_ds_on_. As shown in Figure 6c, the conventional DVCC cannot measure a voltage less than −1.7 V, while the improved DVCC solves this problem (see Figure 6d). Table 3 selects three measurement points, which are located at the moments when the first, second, and third negative peaks of the drain–source voltage of the DUT occur, to compare the *v*_ds_ (actual value) and the output voltage *v*_out_. As shown in Table 3, during the on-oscillation state, the max voltage measurement relative error of the conventional DVCC can reach up to 78.8%, while the error value of the improved DVCC is reduced to less than 17.6%. The comparison results of *v*_ds_ (actual value) and *v*_out_ during the on-steady state are shown in Table 4, which shows that the relative errors of the on-steady state voltage measured by the conventional DVCC and the improved DVCC are both within 1%. Furthermore, the conduction loss measurement errors of the conventional DVCC and the improved DVCC, as shown in Table 5, are 6.42% and 0.78%, respectively, which proves the high accuracy of the conduction loss measurement of the improved circuit.

**Figure 7:**
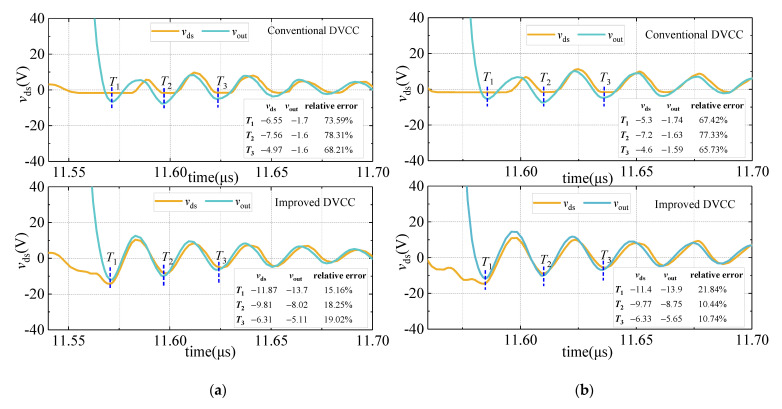
*v*_ds_ and *v*_out_ waveform comparison during the on-oscillation state. (**a**) *V*_DC_ = 400 V; (**b**) *V*_DC_ = 600 V.

To more powerfully illustrate the reduction of conduction loss and on-state voltage measurement error, simulations under different voltages are supplemented. During the test, the *V*_DC_ is set to 400 V and 600 V, respectively, and the measurement results are shown in Figure 7.

The relative error between *v*_ds_ (actual value) and *v*_out_ during the on-oscillation state is shown in Figure 7. Compared with the conventional DVCC, the measurement relative error of the improved DVCC is significantly reduced. When *V*_DC_ is 400 V and 600 V, the maximum relative errors are reduced from 78.31% and 77.33% to 19.02% and 21.84%, respectively. Furthermore, by comparing the conduction loss value measured by the conventional DVCC and the improved DVCC, it can be known that when the *V*_DC_ is 400 V and 600 V, the relative errors are reduced from 6.60% and 6.85% to 1.07% and 1.65%, respectively, which is shown in Table 6.

#### 4.1.2. Qualitative Experimental Verification

**Figure 8:**
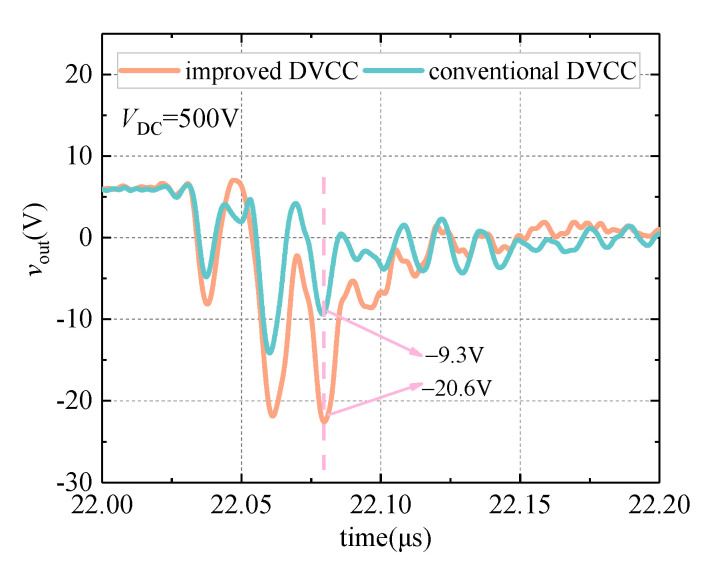
*v*_out_ waveform comparison during the on-oscillation state.

The comparative experiment of the on-oscillation state voltage measurement is carried out in this section. Under the same working condition, the conventional DVCC and improved DVCC are used to measure the on-state voltage of the DUT, respectively. The voltage negative overshoot measured by the two circuits is compared, and the result is shown in Figure 8.

It can be seen from Figure 8 that compared to the improved DVCC, the drain–source voltage negative overshoot measured by the conventional DVCC is significantly reduced. Under the test voltage conditions of 500 V, the measurement result of the voltage negative overshoot is reduced by 11.3 V, which is consistent with the theoretical analysis in Section 2.2.1 and the simulation verification in Section 4.2.1.

### 4.2. Working Security of Auxiliary Device MOSFET

#### 4.2.1. Comparison of Work Security of M in Conventional DVCC and Improved DVCC

**Figure 9:**
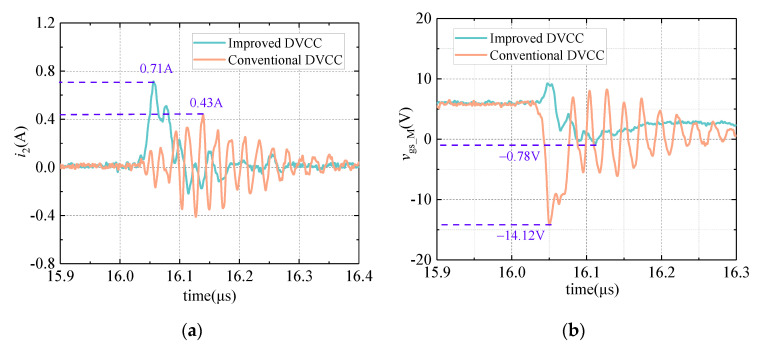
The current waveform flowing through *V*_cc_ and the gate−source voltage waveform of M. (**a**) Current waveform; (**b**) voltage waveform.

The experiments are carried out utilizing the test platform shown in Figure 4, with the primary circuit (DPTC) operating conditions unchanged. Due to the presence of the gate-shunt capacitance, the gate–source voltage negative overshoot of M may exceed its tolerance limit. Therefore, the *V*_DC_ is set to 400 V to ensure the safety of M. The current waveform flowing through *V*_cc_ and the gate−source voltage waveform of M are shown in Figure 9.

The improved DVCC reduces the absolute value of *V*_gs_M(max)_ from 14.12 to 0.78 V but increases the max current flowing through *V*_cc_ from 0.43 to 0.71 A. As seen, the increase in current can be ignored because it is still far less than the impulse tolerance of the DC voltage supply. However, according to the datasheet, the gate−source withstand voltage limit *V*_gs_limit_ of M is only −10 V. Therefore, the reduction of *V*_gs_M(max)_ from −14.12 to −0.78 V makes M out of unsafe conditions, which significantly improves the work security of M.

#### 4.2.2. Work Security of M and *V*_cc_ at Higher Voltages

**Figure 10:**
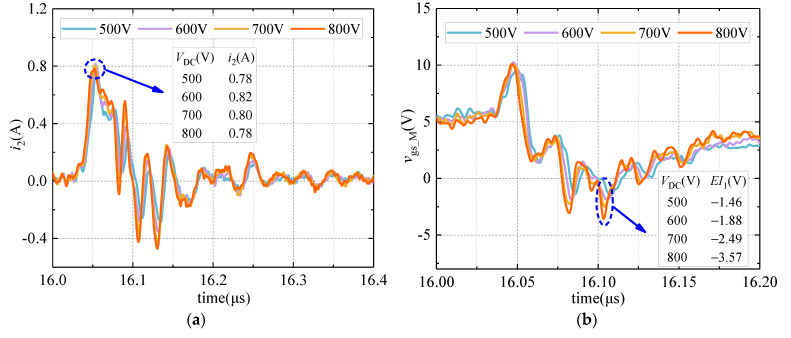
The current waveform flowing through *V*_cc_ and the gate−source voltage waveform of M under higher voltages. (**a**) Current waveform; (**b**) voltage waveform.

To study the working safety of *V*_cc_ and M in the improved DVCC under higher voltages (500 V, 600 V, 700 V, 800 V), related experiments are carried out. The circuit components are selected based on the selection theory proposed in this article to ensure the safety of the experiments. Since the target maximum experimental voltage is 800 V, *V*_D3_ is set to 7.5 V, *V*_cc_ is set to 7 V, *R*_2_ is selected to 50 Ω, and *R*_3_ is selected to 5 Ω for the experiment. The results are shown in Figure 10.

As shown in Figure 10, at higher voltages, the gate–source voltage negative overshoot of M (*EI*_1_) is always within the maximum rating of the gate–source voltage of M. In addition, at the moment of turning off, the maximum current flowing through the DC source (*V*_cc_) is about 0.8 A, which is much smaller than the impulse current tolerance of the DC source. Therefore, the improved circuit proposed in this article can still work effectively and safely under high-voltage conditions.

### 4.3. Selection Method of V_cc_ and V_D3_

**Figure 11:**
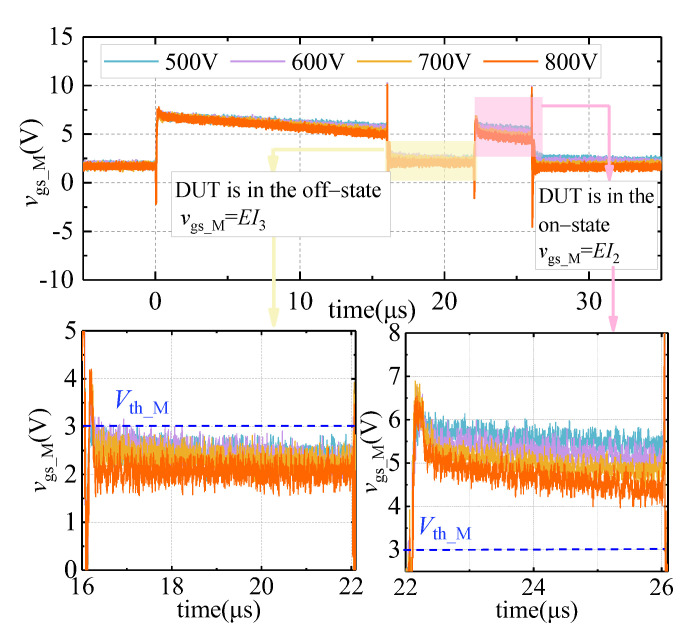
The gate-source voltage waveform of M under different voltages (*V*_cc_ = 7 V, *V*_D3_ = 7.5 V).

According to the working principle of the double pulse circuit and the device parameters of the DUT, when the off-state voltage of the DUT is 800 V, the maximum on-state voltage *V*_on_max_ can reach 3.2 V. Based on the selection mechanism and detailed analyses in Section 3, set *V*_cc_ to 7 V and *V*_D3_ to 7.5 V for the experiment. The results are depicted in Figure 11.

As shown in Figure 11, under different voltages, when the DUT is in the on state, it always meets *EI*_2_ > *V*_th_M_, ensuring that M is normally turned on. When the DUT is in the off state, it satisfies *EI*_3_ < *V*_th_M_, so that M is also in the off state. The experimental results prove the rationality of the selection method of *V*_cc_ and *V*_D3_.

### 4.4. Selection Method of R_2_ and R_3_

**Figure 12:**
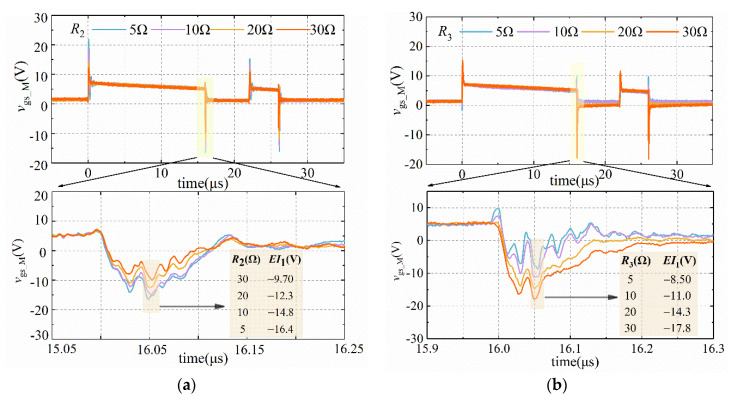
The variation of *EI*_1_ with *R*_2_ and *R*_3_. (**a**) With *R*_2_; (**b**) with *R*_3_.

According to Equations (28) and (31), *R*_3_ > 1.52 Ω and *R*_2_ < 55 Ω while meeting SSC and MEC. Using the controlled variable method, when *V*_cc_, *V*_D3_, and *R*_3_ are determined, set *R*_2_ to 5 Ω, 10 Ω, 20 Ω, and 30 Ω for the experiments. Figure 12a demonstrates the measured *EI*_1_ in the case of different *R*_2_. Moreover, when *R*_2_ is determined, set the resistance of *R*_3_ to 5 Ω, 10 Ω, 20 Ω, and 30 Ω for the experiments. The variation of *EI*_1_ with *R*_3_ is shown in Figure 12b.

As described in Figure 12, *EI*_1_ decreases with the increase in *R*_2_, and it increases with the increase in *R*_3_. The experimental results effectively verify the correctness of the theoretical analysis indicated in Section 3. When choosing *R*_2_, the resistance should be as large as possible while meeting SSC. In contrast, when choosing *R*_3_, the resistance should be as small as possible while completing MEC.

### 4.5. Error Analysis

**Figure 13:**
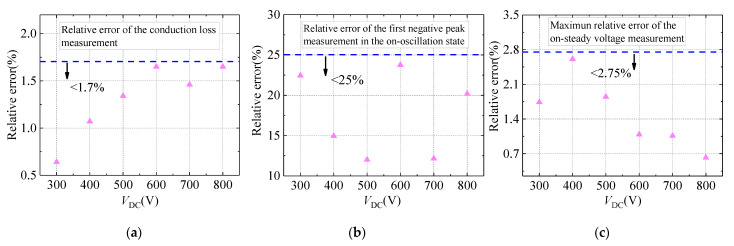
The measurement relative error of improved DVCC. (**a**) Conduction loss; (**b**) on-oscillation state voltage; (**c**) on-steady state voltage.

The size of the error in on-state voltage and conduction loss measurement, using the improved DVCC, is further analyzed to highlight the accuracy over different working conditions. Based on the circuit shown in Figure 5, the on-state voltage of the DUT is measured by the improved DVCC under the *V*_DC_ of 300 V, 400 V, 500 V, 600 V, 700 V, and 800 V. Subsequently, utilizing the measured on-state voltage, the conduction loss is calculated. Furthermore, the measurement results of improved DVCC are compared with the actual value to get the relative error, which is shown in Figure 13.

It can be seen from Figure 13 that when using the improved DVCC to measure the conduction loss of the DUT, the relative error between the measurement result and the actual value remains below 1.7%. In addition, at the moment of turning on, the measurement relative error of the first negative peak is kept within 25%. Moreover, in the on-steady state, the maximum relative error of the on-steady state voltage measurement is within 2.75%. The above data effectively prove the high measurement accuracy of the improved DVCC.

## 5. Conclusions

An improved DVCC topology for measuring the conduction loss of power semiconductor devices is proposed and fully characterized in this article. The proposed DVCC, in comparison with the existing designs (conventional DVCC) through simulation and experimentation, shows better accuracy and higher security. During the on-oscillation state, the maximum relative error of the on-state voltage measurement decreased from 78.8% to 17.6%, and the on-state voltage measurement accuracy is greatly improved. Furthermore, the relative error of the total conduction loss measurement of the two on-state stages is reduced from 6.42% to 0.78%, which is one of the critical contributions of the proposed approach. Another key advantage of the improved DVCC is that it improves the working security of M, which is embodied in the reduction of the gate−source voltage negative overshoot of the auxiliary device MOSFET from −14.12 to 0.78 V. In addition, the influence of component parameters on the circuit performance of the improved DVCC is discussed, and three electrical quantities are extracted as the judgment indicators for the component selection, including the gate−source voltage negative overshoot (*V*_gs_M(max)_) of M, the gate−source voltage *v*_gs_M(on)_ of M when the DUT is in the on state, and the gate−source voltage *v*_gs_M(off)_ of M when the DUT is in the off state. Finally, the component selection criteria are given and validated by experimental results. First, the switching speed and blocking voltage level of M should be consistent with or better than the DUT. Second, in the case of meeting VCEW, the breakdown voltage of the Zener diode (*D*_3_) should be selected to be a small value. Third, under the conditions of fulfilling MEC and SSC, the selection guide for these two resistors is to increase *R*_2_ and decrease *R*_3_ as much as possible.

## Figures and Tables

**Table 1:** Test conditions.

Parameters	Value
*V*_DC_	400 V/500 V
*C*_bus_	200 μF
*L*_load_	0.7 mH
*V*_g_	+20 V/−5 V

**Table 2:** Circuit parameter index.

Symbol	Parameters
*L*_g1_	Parasitic inductance of the gate of the DUT
*L*_d1_	Parasitic inductance of the drain of the DUT
*L*_s1_	Parasitic inductance of the source of the DUT
*R*_d1_	Parasitic resistance of the drain of the DUT
*R*_s1_	Parasitic resistance of the source of the DUT
*R*_g_	Gate drive resistance of the DUT
*D*_0_	Freewheeling diode
*V*_DC_	Bus voltage

**Table 3:** Comparison of *v*_ds_ and *v*_out_ during the on-oscillation state.

Time	Circuit	*v*_ds_/V	*v*_out_/V	Relative Error
*T*_1_	Conventional	−6.9	−1.7	75.4%
	Improved	−14.3	−15.3	7.0%
*T*_2_	Conventional	−8.0	−1.7	78.8%
	Improved	−10.3	−8.5	17.5%
*T*_3_	Conventional	−5.6	−1.7	77.0%
	Improved	−7.4	−6.1	17.6%

**Table 4:** Comparison of *v*_ds_ and *v*_out_ during the on-steady state.

Time	Circuit	*v*_ds_/V	*v*_out_/V	Relative Error
*T*_4_	Conventional	1.710399	1.717680	0.43%
	Improved	1.715286	1.724388	0.53%
*T*_5_	Conventional	1.753108	1.752805	0.02%
	Improved	1.752987	1.752228	0.04%
*T*_6_	Conventional	1.800825	1.800813	0.0007%
	Improved	1.800945	1.800930	0.0009%

**Table 5:** Comparison of conduction loss measurement.

Circuit	Measure Directly/μJ	Measure by DVCC/μJ	Relative Error
Conventional	35.733	38.027	6.42%
Improved	37.010	36.721	0.78%

**Table 6:** Comparison of conduction loss measurement under different voltages.

Voltage	Circuit	Measure Directly/μJ	Measure by DVCC/μJ	Relative Error
400 V	Conventional	22.40	23.87	6.60%
	Improved	23.32	23.07	1.07%
600 V	Conventional	52.92	56.55	6.85%
	Improved	54.52	53.62	1.65%

## Data Availability

The data used for the manuscript are available for researchers on request.

## References

[1] Zhao Z.M., Shi B.C., Zhu Y.C. (2019). Control Technologies for Power Electronic Hybrid Systems in High-voltage High-power Applications: A Review. High Volt. Eng..

[2] Lagier T., Ladoux P., Dworakowski P. (2017). Potential of silicon carbide MOSFETs in the DC/DC converters for future HVDC offshore wind farms. High Volt..

[3] Gareau J., Hou R., Emadi A. (2020). Review of Loss Distribution, Analysis, and Measurement Techniques for GaN HEMTs. IEEE Trans. Power Electron..

[4] Fuentes C.D., Müller M., Bernet S., Kouro S. (2021). SiC-MOSFET or Si-IGBT: Comparison of Design and Key Characteristics of a 690 V Grid-Tied Industrial Two-Level Voltage Source Converter. Energies.

[5] Cai Y.M., Zhao Z.B., Liang S., Sun P., Yang F. (2021). Influence of Parasitic Parameters of Commutation Path on Switching Characteristics of Silicon Carbide MOSFET. High Volt. Eng..

[6] Sun P., Zhao Z., Cai Y., Ke J., Cui X., Ji B. (2020). Analytical model for predicting the junction temperature of chips considering the internal electrothermal coupling inside SiC metal-oxide-semiconductor field-effect transistor modules. IET Power Electron..

[7] Chen M., Hu A., Tang Y., Wang B. (2011). Modeling Analysis of IGBT Thermal Model High Voltage Engineering. High Volt. Eng..

[8] Lei W.J., Liu J.J., Lv G.T., Lv C.L., Cao R. (2020). Review of Reliability Comprehensive Analysis and Evaluation Methods for Key Components and System of Large Capacity Power Electronic Equipment. High Volt. Eng..

[9] Ladoux P., Blaquiere J.M., Alvarez S., Carroll E., Streit P. Test bench for the characterisation of experimental low voltage IGCTs. Proceedings of the 35th Annual IEEE Power Electronics Specialists Conference (PESC 04).

[10] Anurag A., Acharya S., Bhattacharya S. (2020). An Accurate Calorimetric Loss Measurement Method for SiC MOSFETs. IEEE J. Emerg. Sel. Top. Power Electron..

[11] Li H., Li X., Zhang Z., Wang J., Liu L., Bala S. A Simple Calorimetric Technique for High-Efficiency GaN Inverter Transistor Loss Measurement. Proceedings of the 5th IEEE Workshop on Wide Bandgap Power Devices and Applications (WiPDA).

[12] Johannesson D., Nawaz M. Assessment of PSpice Model for Commercial SiC MOSFET Power Modules. Proceedings of the 3rd IEEE Workshop on Wide Bandgap Power Devices and Applications (WiPDA).

[13] Hsin-Ju C., Kusic G.L., Reed G.F. Comparative PSCAD and Matlab/Simulink simulation models of power losses for SiC MOSFET and Si IGBT devices. Proceedings of the 2012 IEEE Power and Energy Conference at Illinois.

[14] Prasad J.S.S., Prasad K.N.V., Narayanan G. Device Loss and Thermal Characteristics of High Power PWM Converters. Proceedings of the 8th IEEE India International Conference on Power Electronics (IICPE).

[15] Lim H., Hwang J., Kwon S., Baek H., Uhm J., Lee G. (2021). A Study on Real Time IGBT Junction Temperature Estimation Using the NTC and Calculation of Power Losses in the Automotive Inverter System. Sensors.

[16] Ahmed M.H., Wang M., Hassan M.A.S., Ullah I. (2019). Power Loss Model and Efficiency Analysis of Three-Phase Inverter Based on SiC MOSFETs for PV Applications. IEEE Access.

[17] Zhang L., Yuan X., Wu X., Shi C., Zhang J., Zhang Y. (2019). Performance Evaluation of High-Power SiC MOSFET Modules in Comparison to Si IGBT Modules. IEEE Trans. Power Electron..

[18] Gelagaev R., Jacqmaer P., Driesen J. (2015). A Fast Voltage Clamp Circuit for the Accurate Measurement of the Dynamic ON-Resistance of Power Transistors. IEEE Trans. Ind. Electron..

[19] Carsten B. Clipping preamplifier provides accurate measurement of transistor conduction voltages. Proceedings of the 31st International Power Conversion Electronic Conference Exhibition.

[20] Pokryvailo A., Carp C. Accurate Measurement of on-State Losses of Power Semiconductors. Proceedings of the Proceedings of the 2008 IEEE International Power Modulators and High Voltage Conference.

[21] Ren L., Shen Q., Gong C. A Voltage Clamp Circuit for the Real-Time Measurement of the On-State Voltage of Power Transistors. Proceedings of the 8th Annual IEEE Energy Conversion Congress and Exposition (ECCE).

[22] Ghimire P., de Vega A.R., Beczkowski S., Munk-Nielsen S., Rannested B., Thogersen P.B. An online V-ce measurement and temperature estimation method for high power IGBT module in normal PWM operation. Proceedings of the International Power Electronics Conference (IPEC-ECCE-ASIA).

[23] Bęczkowski S., Ghimre P., de Vega A.R., Munk-Nielsen S., Rannestad B., Thøgersen P. Online Vce measurement method for wear-out monitoring of high power IGBT modules. Proceedings of the 2013 15th European Conference on Power Electronics and Applications (EPE).

[24] Yu B., Wang L., Ahmed D. (2020). Drain-Source Voltage Clamp Circuit for Online Accurate ON-State Resistance Measurement of SiC MOSFETs in DC Solid-State Power Controller. IEEE J. Emerg. Sel. Top. Power Electron..

[25] Guacci M., Bortis D., Kolar J.W. (2018). On-state voltage measurement of fast switching power semiconductors. CPSS Trans. Power Electron. Appl..

[26] Li R., Wu X., Yang S., Sheng K. (2019). Dynamic on-State Resistance Test and Evaluation of GaN Power Devices Under Hard- and Soft-Switching Conditions by Double and Multiple Pulses. IEEE Trans. Power Electron..

[27] Lu B., Palacios T., Risbud D., Bahl S., Anderson D.I. Extraction of Dynamic On-Resistance in GaN Transistors: Under Soft- and Hard-Switching Conditions. Proceedings of the 2011 IEEE Compound Semiconductor Integrated Circuit Symposium (CSICS).

[28] Wang J., Chung H.S.-H., Li R.T.-H. (2013). Characterization and Experimental Assessment of the Effects of Parasitic Elements on the MOSFET Switching Performance. IEEE Trans. Power Electron..

[29] Ke J.J., Zhao Z.B., XIe Z.K., Xu P., Cui X. (2018). Analytical Switching Transient Model for Silicon Carbide MOSFET under the Influence of Parasitic Parameters. Trans. China Electrotech. Soc..

[30] Gelagaev R., Jacqmaer P., Everts J., Driesen J. A Novel Voltage Clamp Circuit for the Measurement of Transistor Dynamic On-Resistance. Proceedings of the 29th Annual IEEE International Instrumentation and Measurement Technology Conference (I2MTC).

